# Viral hepatitis moderates the impact of TGFB1 on neurocognitive impairment

**DOI:** 10.1002/kjm2.12872

**Published:** 2024-07-06

**Authors:** Wei‐Chia Tsao, Rwei‐Ling Yu, Chi‐Ting Li, Wei‐Fang Tsai, Wan‐Long Chuang, Jee‐Fu Huang, Chia‐Yen Dai, Chun‐Hsiang Tan

**Affiliations:** ^1^ Department of Neurology Kaohsiung Medical University Hospital, Kaohsiung Medical University Kaohsiung Taiwan; ^2^ Institute of Behavioral Medicine, College of Medicine National Cheng Kung University Tainan Taiwan; ^3^ Department of Psychology Kaohsiung Medical University Kaohsiung Taiwan; ^4^ Graduate Institute of Clinical Medicine, College of Medicine Kaohsiung Medical University Kaohsiung Taiwan; ^5^ Hepatobiliary Division, Department of Internal Medicine, and Hepatitis Center Kaohsiung Medical University Hospital Kaohsiung Medical University Kaohsiung Taiwan

**Keywords:** cognitive function, hepatitis, inflammation, polymorphism, TGFB1

## Abstract

Recent studies have identified a correlation between chronic viral hepatitis and cognitive impairment, yet the underlying mechanisms remain unclear. This study investigated the influence of TGFB1 genetic polymorphisms on cognitive function in individuals with and without hepatitis infections, hypothesizing that these polymorphisms and the viral hepatitis‐induced inflammatory environment interact to affect cognitive abilities. Participants (173 with viral hepatitis and 258 healthy controls) were recruited. Genotyping of TGFB1 SNPs was performed using the C2‐58 Axiom Genome‐Wide TWB 2.0 Array Plate. Cognitive function was assessed using the MMSE and MoCA tests. Our results showed that healthy individuals carrying the C allele of rs2241715 displayed better performance in sentence writing (*p* = 0.020) and language tasks (*p* = 0.022). Notably, viral hepatitis was found to moderate the impact of the rs2241715 genotype on language function (*p* = 0.002). Similarly, those carrying the T allele of rs10417924 demonstrated superior orientation to time (*p* = 0.002), with viral hepatitis modifying the influence of the SNP on this particular cognitive function (*p* = 0.010). Our findings underscore the significant role of TGFβ1 in cognitive function and the moderating impact of viral hepatitis on TGFB1 SNP effects. These findings illuminate the potential of TGFB1 as a therapeutic target for cognitive impairment induced by viral hepatitis, thus broadening our understanding of TGFβ1 functionality in the pathogenesis of neurodegeneration.

**Abbreviations**
HCC: hepatocellular carcinoma
MMSE: mini‐mental state examination
MoCA: Montreal Cognitive Assessment
SNP: single‐nucleotide polymorphism
TGFB1: transforming growth factor beta 1

## 1 INTRODUCTION

Recent epidemiological studies have highlighted a link between chronic viral hepatitis and cognitive impairment. Studies, such as one analyzing health insurance data from Taiwan, demonstrated a significantly increased hazard ratio of dementia in the hepatitis C virus (HCV)‐infected population, even after adjusting for conventional risk factors.^1^ Similarly, a South Korean national cohort study observed a higher HCV infection rate in dementia patients.^2^ Our previous work, using comprehensive neuropsychological tests, identified specific cognitive impairment profiles attributable to hepatitis B virus (HBV) or HCV infections.^3^ An examination of neuropsychological performance in HCV and HBV patients revealed verbal learning and memory deficits in both groups.^4^ The mechanisms underlying these associations, possibly involving direct viral infection, activation of intracerebral immune responses, or circulating cytokines, remain elusive.

Persistent effects on the immune system may occur even after virus elimination or replication suppression. While initially a defense mechanism, chronic inflammation can be detrimental if uncontrolled. Studies have linked cognitive dysfunction to inflammatory cytokine levels, such as elevated midlife interleukin‐6 levels predicting cognitive decline.^5^ Continuous cytokine release may persist years after HCV infection,^6^ potentially altering immune cell expression, function, and phenotype. This suggests that viral hepatitis‐associated cognitive impairment may be inflammation‐mediated.^7^

Our past research indicated that *TGFB1* polymorphisms could influence hepatitis patients' inflammation levels,^8^ a finding supported by a recent meta‐analysis showing the significant impact of *TGFB1* polymorphisms on the incidence of cirrhosis among individuals with HCV.^9^ *TGFB1* gene region polymorphisms can also alter TGFβ1 transcription, expression, and function, affecting viral infection susceptibility and disease progression.^10^ The ‐509C>T (rs1800469) polymorphism, for instance, is associated with higher TGFβ1 expression and a slightly increased risk of Alzheimer's disease (AD) progression in Caucasians.^11^ Conversely, the Leu10Pro (rs1800470) polymorphism, linked to increased TGFβ1 secretion,^12^ is associated with reduced vascular dementia and cerebral amyloid angiopathy risk in Japanese‐Americans.^13^ Additionally, a Brazilian study found that the +25G>C (rs1800471) polymorphism, associated with lower TGFβ1 production,^14^ was more common in individuals with cognitive impairment, supporting the association between lower TGFβ1 levels and AD risk.^15^ Despite existing knowledge, few studies have examined *TGFB1*'s role in viral hepatitis‐associated cognitive impairment.

This study aims to investigate the impact of *TGFB1* polymorphism on cognitive function in individuals with and without hepatitis infections. It is hypothesized that the interaction between *TGFB1* genetic polymorphisms and the viral hepatitis‐induced inflammatory microenvironment may lead to altered inflammatory responses, resulting in different levels of cognitive impairment.

## 2 MATERIALS AND METHODS

### 2.1 Participants

Patients with hepatitis B and C infections were enrolled from neurology and hepatobiliary clinics at Kaohsiung Medical University Hospital from 2017 to 2021. Healthy controls were also recruited from the same period's volunteers. HBV diagnosis was confirmed with positive serum HBsAg tests, while HCV diagnosis required positive serum anti‐HCV IgG tests. We collected demographic and clinical characteristics, including age, sex, and education years, through interviews and medical records. Exclusion criteria included severe cerebrovascular disease, brain surgery, thyroid disorder, untreated malignancy, alcohol abuse, illicit drug use, HIV infection, chronic kidney disease (estimated glomerular filtration rate < 30 mL/min/1.73 m^2^), decompensated cirrhosis, and disabling psychiatric disorders. Decompensated cirrhosis was assessed based on history and clinical evaluation. The final statistical analysis included 173 individuals with viral hepatitis and 258 controls, with one missing data point in the control group for rs2241715 genotyping.

### 2.2 Genotyping

Genomic DNA was extracted from the peripheral blood leukocytes of participants. Genotyping of *TGFB1* SNPs employed the C2‐58 Axiom Genome‐Wide TWB 2.0 Array Plate (Affymetrix GeneChip platform). Data analysis utilized PLINK 1.09 beta. SNPs with a minor allele frequency (MAF) < 10% were excluded. A total of 22 SNP loci of the *TGFB1* gene were analyzed. The genotype distributions for all SNPs in the study adhered to Hardy–Weinberg equilibrium among the study groups, as assessed by chi‐square analysis. Pairwise linkage disequilibrium (LD) and haplotype frequencies among the SNPs were examined using Haploview (Broad Institute of MIT and Harvard, version 4.2).

### 2.3 Cognitive measures

Cognitive assessments for all participants were conducted using the traditional Chinese versions of the MMSE and MoCA, which have demonstrated robust validity and reliability in numerous studies.^16^ The MMSE evaluates orientation, attention, recall, language, visual construction, and comprehension. In contrast, the MoCA assesses memory, orientation, attention, language, visual construction, naming, and abstract concepts. The MMSE and MoCA tests used to evaluate major neurocognitive domains are summarized in Table 1.

**TABLE 2:** Comparison of baseline characteristics between hepatitis and controls.

	Ctrl	Hepatitis	Statistic	Total
	*n* = 258	*n* = 173	*p*‐value	*n* = 431
Sex				
Male	69	84	**<0.001**	153
Female	189	89		278
Age (years)				
Mean ± SD	65.11 ± 7.036	61.80 ± 7.475	**<0.001**	63.78 ± 7.388
Education (years)				
Mean ± SD	12.32 ± 3.995	11.69 ± 3.811	0.101	12.07 ± 3.930
Cognition				
MMSE	27.11 ± 2.737	27.38 ± 2.397	0.304	27.22 ± 2.607
MoCA	24.91 ± 3.849	24.43 ± 3.807	0.197	24.72 ± 3.835

*Note*: Bold vlaue indicates statistically significance *p* < 0.025.

### 2.4 Statistical analysis

All statistical analyses were performed using IBM SPSS Statistics for Windows (Version 22.0). After applying the Bonferroni correction (for studying two SNPs, *p* = 0.05/2 = 0.025), a significance level of *p* < 0.025 was set. Quantitative variables were expressed as means ± standard deviation, and qualitative variables as percentages. Moderation analysis used the PROCESS (version 3.5),^17^ incorporating hepatitis infection status as the moderator variable (*W*), the total and subscores of the MMSE and MoCA as outcome variables (*Y*), and *TGFB1* SNPs (rs2241715, rs10417924) as independent variables (*X*). The analyses adjusted for age, sex, and educational years.

## 3 RESULTS

### 3.1 Participant characteristics

The study included 431 participants: 173 with viral hepatitis and 258 healthy controls. Among those with viral hepatitis, 63 were HBV‐positive, 100 were HCV‐positive, and 10 had both infections. Among the HCV‐positive participants, the treatment regimens and their outcomes are detailed in Tables S1 and S2. Seventy two achieved sustained virologic response (SVR), and 28 did not achieve SVR. Specifically, 52.8% (38/72) treated with Peginterferon, 43.1% (31/72) with direct‐acting antivirals (DAAs), and 4.1% (3/72) with both DAAs and Peginterferon achieved SVR, while 96.4% (27/28) of the participants who did not achieve SVR were treatment‐naive, and 3.6% (1/28) of the participants had received Peginterferon without achieving SVR. For the 10 participants with HBV and HCV coinfection, all five participants who achieved SVR were treated with DAAs, while the five participants in the non‐SVR group were all treatment‐naive. Participant demographics are detailed in Table 2. The cohort comprised 153 men and 278 women, with an average education of 12.07 ± 3.930 years. The overall mean age was 63.78 ± 7.388 years, with the healthy control group (65.11 ± 7.036 years) being older than the hepatitis group (61.80 ± 7.475 years) (*p* < 0.001). Cognitive assessment results using MMSE and MoCA are summarized in Table 2, indicating mean scores of 27.11 ± 2.737 and 27.38 ± 2.397 for MMSE and 24.91 ± 3.84 and 24.43 ± 3.807 for MoCA in the control and hepatitis groups, respectively.

**TABLE 1:** Cognition domains and their corresponding tests in MMSE and MoCA.

Domain names	MoCA	MMSE
Orientation	Orientation to time and place	Orientation to time Orientation to time place
Attention	Serial 7's Vigilance test for letter “A” Digit span (5 forward, 3 backward)	Registration (3 trials, 3 words) Serial 7's
Memory	Recall (5 words after 5 min delay)	Recall (>10 s delay, 3 words)
Language	Verbal fluency for letter F Repetition (2 longer sentences)	Naming (pencil, watch) Repetition (1 short sentence) Read and obey commands Write a sentence
Naming	Naming (lion, rhinoceros, camel)	—
Visual construction	Copy cube and draw a clock face	Copy intersecting pentagons
Abstract concepts	Abstraction (word similarities)	—
Comprehension	—	Follow 3‐stage command

### 3.2 Genotype and allele frequencies

**FIGURE 1:**
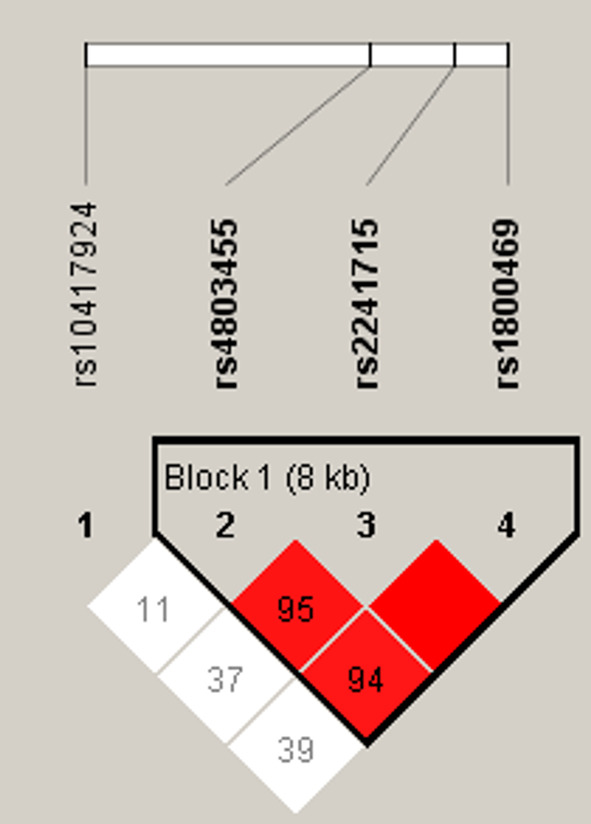
Haploview plot of TGFB1 SNPs analyzed. The relative positions of the SNPs in the TGB1 gene (oriented from right to left, 5′ to 3′) are displayed above the Haploview diagram. Linkage disequilibrium (LD) between the four SNPs examined is shown. Three of the four SNPs form a tight haplotype block spanning approximately 8 kb within the *TGFB1* gene. The relative LD between pairs of *TGFB1* SNPs is represented by the color‐coded squares, with darker red shades indicating stronger LD between any two SNPs. Each number in the square represents the LD value between pairs of SNPs multiplied by 100. The box is empty when the LD value is 1.

Of the 22 *TGFB1* gene loci examined, nine were polymorphic, and 13 were monomorphic (Table 3). Allele frequencies aligned with those reported by the Exome Aggregation Consortium East Asia (ExAC_EAS)^18^ and the Allele Frequency Aggregator (ALFA) project.^19^ Four SNPs (rs10417924, rs4803455, rs2241715, and rs1800469) with minor allele frequencies >10% underwent LD analysis (Figure 1), revealing strong LD among rs2241715, rs1800469, and rs4803455. Among the three SNPs, rs2241715 had the highest minor allele frequency. Therefore, two SNPs, rs10417924 and rs2241715, without LD with each other, were used for further analysis.

**TABLE 3:** SNPs of *TGFB1* and allelic frequency in the control and hepatitis groups.

SNP ID	Position	Minor/Major allele	Most severe consequence^a^	MAF (%)	HWE (*p*‐value)	Population Alt allele frequency			
						Alt allele	Ref allele	ExAC EAS	ALFA total
rs10417924	41327262	T/C	Intron variant	11.302	*X* ^2^ = 0.165 (*p* = 0.684)	C	T	0.880	0.803
rs190566789	41332269	T/C	Missense variant	3.466	*X* ^2^ = 0.019 (*p =* 0.889)	T	C	0.032	0.001
rs13306709	41332382	T/C	Intron variant	2.456	*X* ^2^ = 0.606 (*p =* 0.436)	T	C	0.024	<0.001
rs11466344	41339408	T/C	Intron variant	5.363	*X* ^2^ = 3.054 (*p =* 0.080)	T	C	0.045	0.186
rs2278422	41339853	C/G	Intron variant	2.144	*X* ^2^ = 0.459 (*p =* 0.498)	G	C	0.980	0.296
rs116975930	41345578	A/C	Intron variant	5.162	*X* ^2^ = 1.052 (*p =* 0.305)	A	C	0.063	0.001
rs4803455	41345604	A/C	Intron variant	34.874	*X* ^2^ = 0.065 (*p =* 0.799)	A	C	0.346	0.481
rs2241715	41350981	C/A	5 prime UTR variant	42.649	*X* ^2^ = 1.239 (*p* = 0.266)	C	A	0.421	0.616
rs1800469	41354391	G/A	Intron variant	42.328	*X* ^2^ = 0.557 (*p =* 0.455)	G	A	0.419	0.672

Abbreviations: ALFA, allele frequency aggregator; Alt, alternative; EAS, ExAC East Asians; HWE, Hardy–Weinberg Equilibrium; MAF, minor allele frequency; Ref, reference; SNP, single nucleotide polymorphism.

^a^ The SNP consequence was predicted and described by Ensembl.

### 3.3 Comparison of cognitive function in control and hepatitis groups with different rs2241715 genotypes

Table 4 analyzes demographic characteristics and cognitive function, considering the moderating influence of viral hepatitis. Participants were categorized into healthy controls and individuals with viral hepatitis, further divided by genotype into C allele carriers (CC/CA) and AA genotype carriers. Demographic variables such as age, sex, and education level were comparable among genotype groups within each study subgroup.

**TABLE 4:** Cognition functions of participants in normal and hepatitis groups stratified by rs2241715 genotypes.

Study group	Normal control (*n* = 257)			Hepatitis (*n* = 173)			Moderation analysis	
Genotype	CC/CA (*n* = 172)	AA (*n* = 85)	Statistic *p*‐value	CC/CA (*n* = 109)	AA (*n* = 64)	Statistic *p*‐value	SNP effect, *p*‐value	Hepatitis moderation effect, *p*‐value
Age (years)	65.30 ± 7.251	64.78 ± 6.638	0.579	62.44 ± 7.566	60.70 ± 7.243	0.140		
M/F	49/123	20/65	0.399	57/52	27/37	0.199		
Education (years)	12.34 ± 3.815	12.24 ± 4.362	0.848	11.99 ± 3.479	11.19 ± 4.302	0.184		
MMSE	27.09 ± 2.876	27.15 ± 2.466	0.703	27.29 ± 2.608	27.52 ± 2.000	0.888	0.865	0.515
Orientation to time	4.58 ± 0.837	4.75 ± 0.532	0.165	4.69 ± 0.648	4.78 ± 0.487	0.410	0.057	0.710
Orientation to place	4.09 ± 0.871	4.19 ± 0.748	0.523	4.68 ± 0.607	4.63 ± 0.577	0.437	0.290	0.370
Registration	2.98 ± 0.170	2.98 ± 0.152	0.474	2.96 ± 0.189	3.00 ± 0.000	0.122	0.720	0.192
Serial 7's	4.53 ± 0.805	4.51 ± 0.881	0.953	4.38 ± 0.869	4.31 ± 1.037	0.997	0.827	0.975
Naming	2.00 ± 0.000	2.00 ± 0.000	—	2.00 ± 0.000	2.00 ± 0.000	—	—	—
Repetition	0.96 ± 0.198	0.91 ± 0.294	0.086	0.96 ± 0.189	0.97 ± 0.175	0.850	0.033	0.166
Read and obey command	0.95 ± 0.211	0.95 ± 0.213	0.984	0.97 ± 0.164	0.98 ± 0.125	0.616	0.908	0.511
Write a sentence	0.91 ± 0.283	0.81 ± 0.393	**0.020**	0.87 ± 0.336	0.88 ± 0.333	0.948	0.030	0.067
Memory	2.49 ± 0.761	2.59 ± 0.660	0.380	2.26 ± 0.865	2.38 ± 0.745	0.497	0.369	0.905
Visual construction	0.89 ± 0.314	0.89 ± 0.310	0.912	0.83 ± 0.373	0.89 ± 0.315	0.315	0.720	0.381
Comprehension	2.72 ± 0.730	2.56 ± 0.879	0.111	2.69 ± 0.766	2.70 ± 0.683	0.809	0.107	0.227
MoCA	25.10 ± 3.865	24.54 ± 3.835	0.224	24.43 ± 4.099	24.42 ± 3.280	0.632	0.150	0.260
Orientation	5.71 ± 0.731	5.82 ± 0.467	0.434	5.83 ± 0.606	5.91 ± 0.344	0.389	0.166	0.855
Attention	5.65 ± 0.707	5.55 ± 0.764	0.288	5.58 ± 0.628	5.58 ± 0.686	0.797	0.267	0.343
Memory	2.53 ± 1.745	2.26 ± 1.767	0.251	2.45 ± 1.787	2.22 ± 1.713	0.392	0.154	0.901
Language	2.43 ± 0.765	2.22 ± 0.792	**0.022**	2.12 ± 0.879	2.36 ± 0.721	0.099	**0.024**	**0.002**
Naming	2.65 ± 0.664	2.61 ± 0.725	0.874	2.59 ± 0.760	2.58 ± 0.686	0.716	0.839	0.571
Visual construction	4.35 ± 0.952	4.18 ± 0.941	0.079	4.12 ± 1.160	4.05 ± 0.950	0.233	0.124	0.337
Abstract concepts	1.27 ± 0.801	1.31 ± 0.802	0.663	1.13 ± 0.795	0.98 ± 0.787	0.244	0.614	0.384

*Note*: Bold vlaue indicates statistically significance *p* < 0.025.

The analysis revealed that among healthy controls, C allele carriers outperformed AA genotype carriers in sentence writing (0.91 ± 0.283 vs. 0.81 ± 0.393, *p* = 0.020) and language tasks on the MoCA scale (2.43 ± 0.765 vs. 2.22 ± 0.792, *p* = 0.022). However, no significant differences in cognitive function were observed between genotype groups among individuals with viral hepatitis, suggesting that the rs2241715 polymorphism in *TGFB1* influences language function among healthy controls, but this effect is not evident in individuals with viral hepatitis.

When analyzing the different types of viral hepatitis (HBV, HCV, and HBV and HCV coinfection) separately (Table S3), the data showed similar results of the impacts of the SNP on cognitive functions to the analysis combining all individuals with viral hepatitis. For the HBV group, no significant differences were found between genotype groups. In the HCV group, no significant differences were observed between genotype groups in any cognitive functions. In the HBV and HCV coinfection group, the small sample size limited the statistical power, and no significant differences were noted.

**FIGURE 2:**
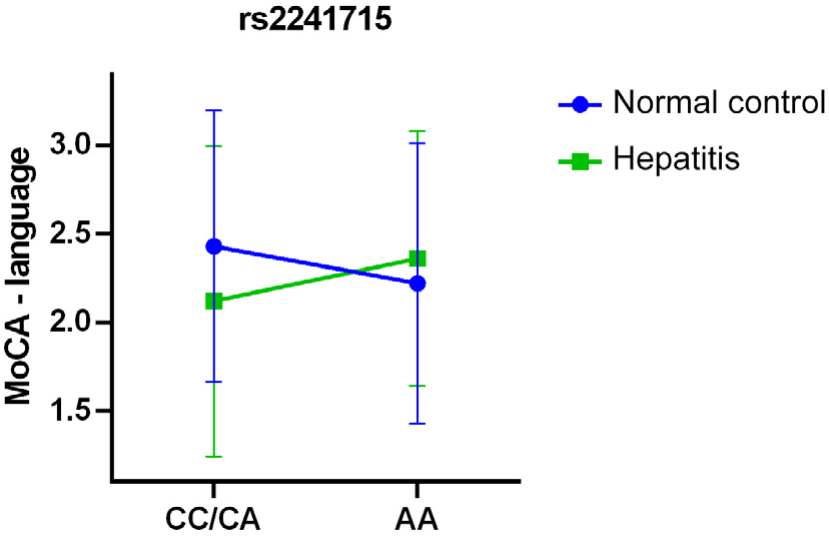
Moderation of the effect of rs2241715 genotypes on MoCA language scores by viral hepatitis. The impact of rs2241715 genotypes on MoCA language scores is moderated by the presence of viral hepatitis. Within the healthy control group, individuals carrying the C allele performed significantly better in language tasks on the MoCA scale than those with the AA genotype (2.43 ± 0.765 vs. 2.22 ± 0.792, *p* = 0.022). However, among individuals with viral hepatitis, the protective effect associated with the C allele is either diminished or even reversed (2.12 ± 0.879 vs. 2.36 ± 0.721, *p* = 0.099).

To evaluate the differential influence of rs2241715 polymorphism on cognitive function between the healthy control group and the viral hepatitis group and to account for potential confounders such as age, sex, and education level, we conducted a moderation analysis based on linear regression, adjusting for these variables (Table 4). The moderation analysis revealed a significant interaction between the rs2241715 polymorphism and viral hepatitis on the language function measured by the MoCA test (*p* = 0.002), in addition to the significant role of the rs2241715 SNP on language function in the MoCA evaluation (*p* = 0.024). Figure 2 provides a graphical representation detailing the significant influence of rs2241715 on language function among the healthy cohort and its diminished impact among individuals with viral hepatitis. The moderation analysis separating the participants into groups of normal control, HBV, HCV, and HBV and HCV coinfection showed similar results to the analysis combining all individuals with viral hepatitis. There was a significant interaction between the rs2241715 polymorphism and viral hepatitis on the language function measured by the MoCA test (*p* < 0.001), in addition to the significant role of the rs2241715 SNP on language function in the MoCA test (*p* = 0.022).

### 3.4 Comparison of cognitive function in control and hepatitis groups with different rs10417924 genotypes

Participants were further divided into subgroups based on rs10417924 genotypes: T allele carriers (TT or TC) and those with the CC genotype (Table 5). Age, sex ratio, and education levels were similar across different genotype groups in both control and hepatitis groups. In the healthy control group, T allele carriers showed significantly better performance in orientation to time compared to those with the CC genotype (4.83 ± 0.606 vs. 4.58 ± 0.786, *p* = 0.002).

**TABLE 5:** Cognition functions of participants in normal and hepatitis groups stratified by rs10417924 genotypes.

Study group	Normal control (*n* = 258)			Hepatitis (*n* = 173)			Moderation analysis	
Genotype	TT/TC (*n* = 64)	CC (*n* = 194)	Statistic *p*‐value	TT/TC (*n* = 40)	CC (*n* = 133)	Statistic *p*‐value	SNP effect, *p*‐value	Hepatitis moderation effect, *p*‐value
Age (years)	65.75 ± 6.749	64.90 ± 7.132	0.401	61.70 ± 8.238	61.83 ± 7.263	0.925		
Male / Female	14/50	55/139	0.310	19/21	65/68	0.879		
Education (years)	12.02 ± 3.982	12.39 ± 3.986	0.519	12.40 ± 4.168	11.48 ± 3.688	0.180		
MMSE	27.64 ± 1.863	26.94 ± 2.953	0.143	27.48 ± 2.364	27.35 ± 2.415	0.580	**0.014**	0.065
Orientation to time	4.83 ± 0.606	4.58 ± 0.786	**0.002**	4.65 ± 0.580	4.74 ± 0.599	0.182	**0.008**	**0.010**
Orientation to place	4.11 ± 0.715	4.12 ± 0.871	0.599	4.58 ± 0.594	4.68 ± 0.595	0.201	0.979	0.418
Registration	3.00 ± 0.000	2.97 ± 0.189	0.248	2.98 ± 0.158	2.98 ± 0.149	0.928	0.194	0.314
Serial 7's	4.70 ± 0.525	4.46 ± 0.900	0.194	4.45 ± 0.904	4.32 ± 0.942	0.373	0.034	0.334
Naming	2.00 ± 0.000	2.00 ± 0.000	—	2.00 ± 0.000	2.00 ± 0.000	—	—	—
Repetition	0.94 ± 0.244	0.94 ± 0.232	0.864	0.93 ± 0.267	0.98 ± 0.149	0.113	0.955	0.206
Read and obey command	0.94 ± 0.244	0.96 ± 0.199	0.485	0.98 ± 0.158	0.98 ± 0.149	0.928	0.494	0.869
Write a sentence	0.89 ± 0.315	0.88 ± 0.330	0.760	0.93 ± 0.267	0.86 ± 0.351	0.260	0.666	0.586
Memory	2.53 ± 0.689	2.52 ± 0.743	0.889	2.30 ± 0.883	2.30 ± 0.807	0.822	0.758	0.624
Visual construction	0.91 ± 0.294	0.89 ± 0.318	0.662	0.88 ± 0.335	0.85 ± 0.359	0.690	0.614	0.884
Comprehension	2.80 ± 0.540	2.62 ± 0.844	0.247	2.83 ± 0.594	2.65 ± 0.769	0.106	0.057	0.644
MoCA	24.67 ± 3.797	24.99 ± 3.873	0.441	25.18 ± 3.012	24.20 ± 3.998	0.279	0.744	0.375
Orientation	5.86 ± 0.467	5.71 ± 0.706	0.090	5.90 ± 0.379	5.84 ± 0.562	0.505	0.067	0.344
Attention	5.66 ± 0.718	5.60 ± 0.729	0.447	5.50 ± 0.641	5.60 ± 0.651	0.255	0.450	0.110
Memory	2.22 ± 1.804	2.51 ± 1.731	0.302	2.88 ± 1.539	2.21 ± 1.797	0.046	0.280	0.036
Language	2.25 ± 0.797	2.40 ± 0.771	0.127	2.28 ± 0.847	2.19 ± 0.827	0.499	0.203	0.390
Naming	2.64 ± 0.698	2.63 ± 0.680	0.868	2.70 ± 0.564	2.55 ± 0.773	0.386	0.608	0.729
Visual construction	4.31 ± 0.924	4.28 ± 0.959	0.857	4.23 ± 0.768	4.05 ± 1.163	0.890	0.493	0.868
Abstract concepts	1.19 ± 0.833	1.31 ± 0.788	0.306	1.10 ± 0.810	1.07 ± 0.790	0.814	0.366	0.803

*Note*: Bold vlaue indicates statistically significance *p* < 0.025.

When analyzing the different types of viral hepatitis separately (Table S4), the data indicated that for the HBV group and the HCV group, no significant differences were found between genotype groups. However, for the HBV and HCV coinfection group, a significant difference was noted in memory tasks (4.00 ± 0.816 for TT/TC vs. 1.50 ± 1.761 for CC, *p* = 0.018), indicating a significant impact of rs10417924 on memory among individuals with HBV and HCV coinfection.

**FIGURE 3:**
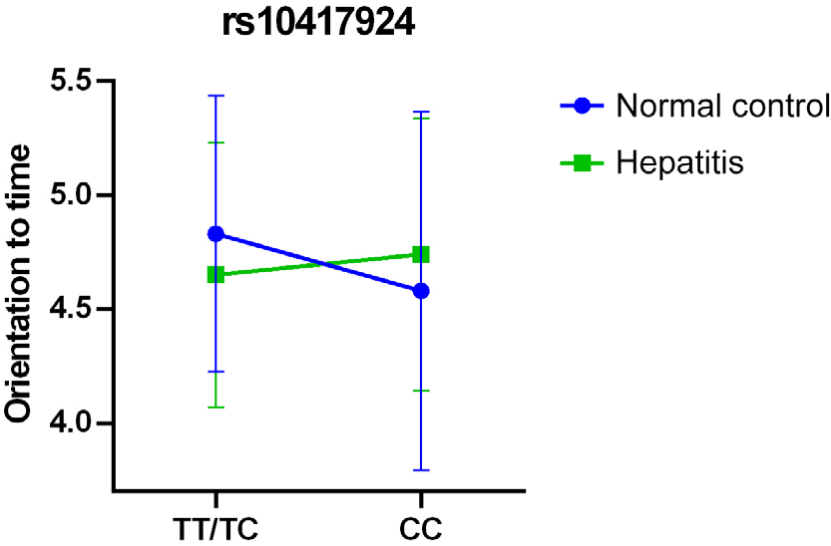
Moderation of the effect of rs10417924 genotypes on orientation to time by viral hepatitis. The impact of rs10417924 genotypes on orientation to time is moderated by the presence of viral hepatitis. Within the healthy control group, individuals carrying the T allele performed significantly better in orientation to time on the MMSE scale than those with the CC genotype (4.83 ± 0.606 vs. 4.58 ± 0.786, *p* = 0.002). However, among individuals with viral hepatitis, the protective effect associated with the T allele is either diminished or even reversed (4.65 ± 0.580 vs. 4.74 ± 0.599, *p* = 0.182).

Moderation analysis revealed a significant rs10417924 polymorphism impact on total MMSE scores (*p* = 0.014). However, the presence of viral hepatitis did not significantly moderate the effect. Additionally, a significant impact of the rs10417924 polymorphism was found on the “orientation to time” task within the MMSE, with a significant interaction between the SNP and viral hepatitis (*p* = 0.010). Figure 3 illustrates the significant influence of rs10417924 on orientation to time among the healthy cohort and its reduced impact among individuals with viral hepatitis, suggesting that the T allele may protect cognitive function, particularly in those without viral hepatitis. The moderation analysis separating the participants into groups of normal control, HBV, HCV, and HBV and HCV coinfection showed similar results to the analysis combining all individuals with viral hepatitis. There was a significant role of the rs10417924 polymorphism in the total MMSE score (*p* = 0.014) and the orientation to time task (*p* = 0.009). However, the significant interaction between the rs10417924 polymorphism and viral hepatitis on orientation to time observed in the combined analysis was not observed when separating the hepatitis groups.

## 4 DISCUSSION

The role of TGFβ in neuronal cell growth, differentiation, and immune balance in the brain is pivotal. TGFβ1 stabilizes Tregs function, inhibits T lymphocyte activation, and induces T helper cell‐mediated inflammation.^20^ It also exhibits neuroprotective effects, such as reducing Aβ burden and inhibiting neuritic plaque formation through promoting microglia‐mediated Aβ degradation.^21^ Additionally, TGFβ signaling inhibits the production of free radicals induced by inflammatory stimuli and induces phagocytosis of Aβ in vitro. However, this induction of phagocytosis is lost with age and in Alzheimer's disease. Impairment of TGFβ signaling can potentiate neuroinflammation, favoring neuronal dysfunction and neurodegenerative changes.^22^ *TGFB1*‐knockout mice showed reduced primary neurons and a thinner cerebral cortex.^23^ Reducing neuronal TGFβ signaling in aged hAPP mice led to Aβ accumulation and dendritic loss.^24^ Conversely, increasing astroglial TGFβ1 reduced amyloid plaques and dystrophic neurites in aged hAPP mice.^21^ In AD patients, decreased plasma TGFβ1 levels^25^ corresponded with the diminished neuroprotective role of TGFβ1.

However, TGFβ1's protective role is not unanimously supported. Chronic TGFβ1 overproduction led to basement membrane protein accumulation, causing AD‐like cerebrovascular amyloidosis and microvascular degeneration.^26^ In a blood–brain barrier (BBB) breakdown animal model, barrier dysfunction triggered TGFβ signaling hyperactivation, leading to an aged brain phenotype. Astrocytic TGFβ receptors’ conditional knockdown or TGFβ signaling pharmacological inhibition reversed aging phenotypes and cognitive impairment in mice.^27^ TgCRND8 mice, a familial AD model, exhibited elevated TGFβ1 levels in cortical brain regions, and adding TGFβ1 to mouse primary cortical neuronal cultures increased apoptosis.^28^ AD patients also showed increased cerebrospinal fluid TGFβ1 levels,^29^ suggesting its detrimental impact on cognitive function.

The contradictory findings, revealing both protective and damaging TGFβ1 roles, suggest its functions in neurodegenerative disease pathogenesis are highly context‐dependent. TGFβ1's dual immunoregulatory properties in the liver, including proinflammatory and anti‐inflammatory responses, are well‐documented. Under normal conditions, TGFβ1 acts as a tumor suppressor and pro‐apoptotic agent in adult hepatocytes, regulating liver mass, while genetic deletion of *TGFB1* in mice on a BALB/c background led to the development of necroinflammatory IFN‐γ‐dependent hepatitis.^30^ However, chronic inflammation disrupts the TGFβ signaling pathway, leading to fibrogenesis, exacerbating liver fibrosis, and escalating hepatocellular carcinoma (HCC) risk. This process results in the accumulation of pro‐metastatic cytokines and genetic alterations in a diseased liver, increasing TGFβ1, which interacts with the surrounding stroma to promote HCC development.^31^

This study primarily aimed to explore the effect of *TGFB1* genetic polymorphism and its interaction with viral hepatitis on cognitive function in individuals with and without hepatitis. Specifically, it sought to elucidate the impact of rs10417924 and rs2241715 polymorphisms on cognitive function while controlling for confounders like age, gender, and education. Our findings indicated that the rs2241715 polymorphism was associated with significant differences in language functions in MMSE and MoCA assessments, and the presence of viral hepatitis moderated its influence. Healthy controls carrying the C allele exhibited better performance in sentence writing and language tasks than AA genotype carriers. The linear regression‐based moderation analysis demonstrated that the presence of viral hepatitis influenced the impact of rs2241715 on cognitive function.

The rs2241715, in linkage disequilibrium with rs1800469 in the present and another IgA nephropathy study,^32^ is located in the *TGFB1* DNase I hypersensitive‐intron 1, a known regulatory region. Previous studies have linked the rs2241715 C allele with increased childhood asthma risk in Chinese populations, while the A allele was associated with better pulmonary function.^33^ As higher TGFβ1 levels correlate with more severe asthma symptoms, it is plausible that individuals carrying the rs2241715 C allele exhibit higher TGFβ1 levels. In our study, control group participants carrying the rs2241715 C allele demonstrated superior performance in MMSE sentence writing and MoCA language sections. However, in the viral hepatitis group, this protective effect of the rs2241715 C allele on language function was not observed, aligning with the aforementioned study and suggesting that the *TGFB1* SNP influences TGFβ1 levels in a specific manner: conferring a protective effect in individuals without viral hepatitis, an effect not observed in those with viral hepatitis.

The rs10417924 SNP, located in the intergenic non‐coding region between the coiled‐coil domain containing 97 (CCDC97) and the *TGFB1* gene,^34^ has received limited attention regarding its impact on cognitive function. However, its physiological significance in various medical conditions is established. For example, it is linked to childhood acute lymphoblastic leukemia risk^35^ and is protective against radiotoxicity in prostate cancer.^36^ Our study found that control group T allele carriers performed better in orientation to time than CC genotype carriers. The linear regression‐based moderation analysis revealed a significant impact of rs10417924 on MMSE total scores and the “orientation to time” task, with viral hepatitis infection status modifying the effect conferred by rs10417924 on cognitive function (in the “orientation to time” task). These results provide additional evidence supporting the significant impact of *TGFB1* SNP on cognitive functions that can be moderated by viral hepatitis infection.

To date, only one study has explored the role of genetic polymorphisms in influencing the impact of hepatitis infection on cognitive function. In this study, the APOE ε4 allele, a well‐known risk locus for AD development, exhibited a protective effect against attention deficit in HCV patients, proposedly due to impaired virus incorporation into central nervous system cells.^37^ Previous research on the correlation between hepatitis and cognitive function demonstrated variable impacts of viral hepatitis on neurodegeneration, even after controlling confounding variables such as comorbid psychiatric conditions, substance abuse, and advanced liver diseases, including cirrhosis, encephalopathy, and HCC.^38^ Our study's findings suggest that this heterogeneity may be partially attributed to the interaction between genetic polymorphisms and inflammation. Moreover, our results demonstrating the influence of *TGFB1* SNPs moderated by viral hepatitis align with previous studies revealing TGFβ1's dual functions in carcinogenesis^31^ and suggest the potential involvement of a similar mechanism in the moderation of cognitive functions and HCC development by *TGFB1* SNPs.

TGFβ1 signaling is a complex network characterized by intricate interactions between upstream regulators and downstream effectors. Key extracellular and intracellular components drive the activation and function of TGFβ1. Central to this pathway, the latency‐associated peptide (LAP) and latent TGFβ‐binding proteins (LTBPs) are instrumental. Within the small latent complex, LAP binds TGFβ, maintaining it in a latent state that precludes interaction with its receptors until activated. LTBPs are essential for embedding latent TGFβ in the extracellular matrix, enabling its activation upon the requisite mechanical or integrin‐mediated distortion of LAP's structure. Activation is also facilitated by proteases such as matrix metalloproteinases and modulators like integrins. These components modify LAP‐LTBP interactions or cleave LAP directly, highlighting the sophisticated regulation of TGFβ activity.^39^, ^40^

Following receptor activation, the signaling cascade within the cell primarily involves Smad proteins. Type I receptor‐mediated phosphorylation of receptor‐activated Smads (R‐Smads) allows their pairing with Smad4. These complexes translocate to the nucleus to influence gene transcription. Additionally, TGFβ signaling's versatility is augmented by interactions with other pathways like MAPK, PI3K, and Rho‐like GTPase, which modulate key cellular processes including proliferation, differentiation, and apoptosis.^40^

Disruptions in TGFβ1 signaling, particularly through its effects on Smad proteins and interactions with pathways such as MAPK and PI3K, have been linked to neurodegenerative diseases and cognitive impairments.^21^ The present study shows that genetic polymorphisms in TGFB1 might significantly affect brain function. In the context of chronic viral hepatitis, systemic inflammation may moderate TGFβ1‐related neuroinflammatory responses and the resultant cognitive functions, providing a mechanistic link between TGFβ1 polymorphisms, viral hepatitis, and altered cognitive functions.

It is important to note that our study population predominantly comprised mature participants, with a mean age of around 63.78. This demographic selection was intentional, aiming to investigate a more prominent decline of cognitive functions with age. This age factor is crucial in interpreting our results, as the decline in cognitive function might be differentially influenced by age in conjunction with chronic hepatitis infection.

It is essential to recognize the limitations of this study. A significant limitation is the absence of an independent cohort for validating our results. Additionally, generalizing these findings to other ethnic groups should be done cautiously due to the distinct genetic backgrounds of the Asian population. Independent replication in another cohort will strengthen the validity of our results. Furthermore, the cross‐sectional design of our study limits our ability to establish a temporal relationship between the viral hepatitis moderation effect on cognitive function progression. Therefore, longitudinal studies are necessary to deepen our understanding of the interplay between *TGFB1* gene polymorphism, inflammation, and cognitive function. Additionally, due to the total number of participants in the present study, the subgrouping of the genotypes was divided into two genotype groups rather than three based on the number of minor alleles. Additionally, serum TGF‐β1 levels were not examined in this study because the interaction between chronic hepatitis infection and TGF‐β1 SNPs to cause differences in cognitive functions is a long‐term process. The successful elimination of HCV or suppression of HBV later in the disease course could disrupt viral activities and consequently cause TGF‐β1 levels at the time of evaluation to differ from the original treatment‐naive state, potentially confounding the results. Furthermore, serum levels may not accurately reflect TGF‐β1 levels in the cerebrospinal fluid and brain, which are more relevant to cognitive function. However, the collection of cerebrospinal fluid from hundreds of participants was beyond the scope of the present study. Future studies recruiting a larger number of participants with measurements of cerebrospinal fluid and serum TGF‐β1 levels may provide further information in differentiating the specific impact of HBV and HCV on moderating the effect of each genotype conferred by *TGFB1* SNPs.

## 5 CONCLUSION

**FIGURE 4:**
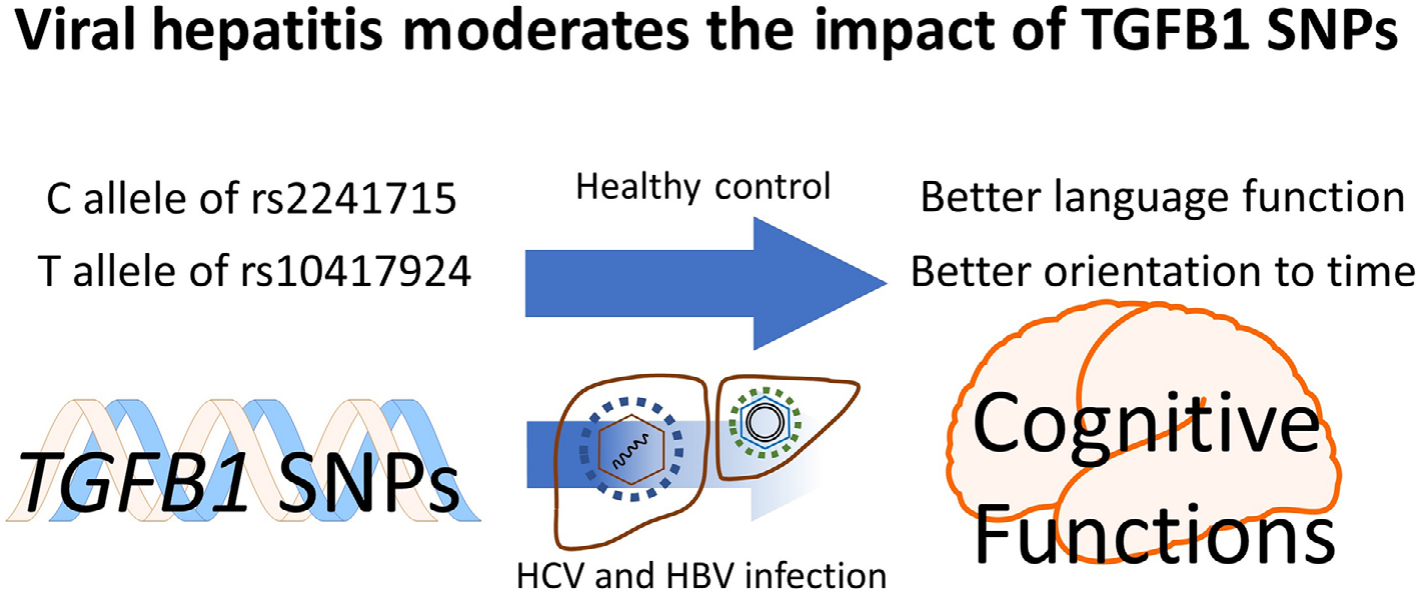
Graphical abstract: Viral hepatitis moderates the impact of TGFB1 SNPs. The study demonstrates that the effect of TGFB1 SNPs on cognitive functions is influenced by viral hepatitis infection status. Specifically, in individuals without viral hepatitis, the rs2241715 polymorphism significantly affects MMSE and MoCA language functions, while the rs10417924 polymorphism influences the “orientation to time” task in the MMSE. However, the presence of viral hepatitis modifies the effects of these SNPs on cognitive functions.

The study demonstrated a significant *TGFB1* SNP impact on cognitive functions moderated by viral hepatitis infection status. The rs2241715 polymorphism significantly impacted MMSE and MoCA language functions, while the rs10417924 polymorphism influenced the “orientation to time” task in the MMSE. The viral hepatitis presence modified these SNPs' effects on cognitive functions (Figure 4). Further research in independent cohorts and larger sample sizes is required to confirm our findings and elucidate the potential underlying mechanisms.

## CONFLICT OF INTEREST STATEMENT

The authors declare no conflict of interest.

## Supporting information


Table S1.–S4.
