# Supplementary material for: Viral hepatitis moderates the impact of TGFB1 on neurocognitive impairment
Source: Kaohsiung J Med Sci. 2024 Jul 6;40(9):852–61. doi: 10.1002/kjm2.12872 (PMC11895573; doi:10.1002/kjm2.12872)
Supplement: Supplementary file 1 — Table S1.–S4. [file KJM2-40-852-s001.docx]

**Supplementary Table 1.** HCV-Specific Treatment Regimens for Chronic HCV Patients

| Treatment regimen | SVR (n = 72) | Non-SVR (n = 28) |
| --- | --- | --- |
| Treatment naïve | - | n =27 (96.4%) |
| Peginterferon | n =38 (52.8%) | n =1　(3.6%) |
| Direct-acting antiviral agents (DAAs) | n =31 (43.1%) | - |
| Both DAAs and Peginterferon | n =3 (4.1%) | - |

**Supplementary Table 2.** HCV-Specific Treatment Regimens for Chronic HBV and HCV Coinfection Patients

| Treatment regimen | SVR (n = 5) | Non-SVR (n = 5) |
| --- | --- | --- |
| Treatment naïve | - | n =5 (100.0%) |
| Peginterferon | - | - |
| Direct-acting antiviral agents (DAAs) | n =5 (100.0%) | - |
| Both DAAs and Peginterferon | - | - |

| **Supplementary Table 3.** Cognition functions of participants in HBV, HCV, and HBV and HCV coinfection groups stratified by rs2241715 genotypes. | | | | | | | | | | | | | | | |
| --- | --- | --- | --- | --- | --- | --- | --- | --- | --- | --- | --- | --- | --- | --- | --- |
| Study Group | Normal Control (n = 257) | | | HBV (n = 63 ) | | | HCV (n = 100 ) | | | HBV + HCV coinfection (n = 10 ) | | | | Moderation analysis | |
| Genotype | CC/CA  (n = 172) | AA  (n = 85) | Statistic *p-value* | CC/CA  (n = 44) | AA  (n = 19) | Statistic  *p-value* | CC/CA  (n = 61) | AA  (n = 39) | Statistic  *p-value* | CC/CA  (n =4) | | AA  (n =6) | Statistic  *p-value* | SNP  effect | Moderation  effect |
| Age (years) | 65.30 ± 7.251 | 64.78 ± 6.638 | p =0.579 | 60.55 ± 6.596 | 59.42 ± 5.326 | *p =0.515* | 63.7 ± 7.79 | 61.62 ± 7.926 | *p =0.199* | 64 ± 11.633 | | 58.83 ± 8.035 | *p =0.426* |  |  |
| M/F | 49 / 123 | 20 / 65 | p =0.399 | 32 / 12 | 12 / 7 | *p =0.552* | 22 / 39 | 12 / 27 | *p =0.668* | 3 / 1 | | 3 / 3 | *p =0.571* |  |  |
| Education (years) | 12.34 ± 3.815 | 12.24 ± 4.362 | p =0.848 | 13.42 ± 3.059 | 12.47 ± 3.85 | *p =0.257* | 10.89 ± 3.474 | 10.26 ± 3.789 | *p =0.229* | 13.00 ± 2.000 | | 13.17 ± 7.278 | *p =0.966* |  |  |
| MMSE | 27.09 ± 2.876 | 27.15 ± 2.466 | p =0.703 | 28.14 ± 1.456 | 27.95 ± 1.393 | p =0.478 | 26.77 ± 3.117 | 27.49 ± 1.833 | p =0.360 | 26.00 ± 1.414 | | 26.33 ± 3.933 | p =0.877 | p =0.865 | p =0.569 |
| Orientation to time | 4.58 ± 0.837 | 4.75 ± 0.532 | p =0.165 | 4.91 ± 0.362 | 4.89 ± 0.315 | p =0.643 | 4.54 ± 0.765 | 4.74 ± 0.549 | p =0.141 | 4.50 ± 0.577 | | 4.67 ± 0.516 | p =0.617 | p =0.058 | p =0.832 |
| Orientation to place | 4.09 ± 0.871 | 4.19 ± 0.748 | p =0.523 | 4.77 ± 0.424 | 4.84 ± 0.375 | p =0.536 | 4.59 ± 0.716 | 4.56 ± 0.598 | p =0.588 | 5.00 ± 0.000 | | 4.33 ± 0.816 | p =0.114 | p =0.294 | p =0.415 |
| Registration | 2.98 ± 0.170 | 2.98 ± 0.152 | p =0.474 | 3.00 ± 0.000 | 3.00 ± 0.000 | - | 2.93 ± 0.25 | 3.00 ± 0.000 | p =0.104 | 3.00 ± 0.000 | | 3.00 ± 0.000 | - | p =0.714 | p =0.328 |
| Serial 7's | 4.53 ± 0.805 | 4.51 ± 0.881 | p =0.953 | 4.55 ± 0.663 | 4.26 ± 0.806 | p =0.173 | 4.26 ± 0.998 | 4.49 ± 0.97 | p =0.162 | 4.25 ± 0.500 | | 3.33 ± 1.633 | p =0.243 | p =0.828 | p =0.095 |
| Naming | 2.00 ± 0.000 | 2.00 ± 0.000 | - | 2.00 ± 0.000 | 2.00 ± 0.000 | - | 2.00 ± 0.000 | 2.00 ± 0.000 | - | 2.00 ± 0.000 | | 2.00 ± 0.000 | - | - | - |
| Repetition | 0.96 ± 0.198 | 0.91 ± 0.294 | p =0.086 | 0.95 ± 0.211 | 1.00 ± 0.000 | p =0.349 | 0.97 ± 0.18 | 0.97 ± 0.16 | p =0.839 | 1.00 ± 0.000 | | 0.83 ± 0.408 | p =0.414 | p =0.030 | p =0.147 |
| Read and obey command | 0.95 ± 0.211 | 0.95 ± 0.213 | p =0.984 | 1.00 ± 0.000 | 0.95 ± 0.229 | p =0.128 | 0.95 ± 0.218 | 1.00 ± 0.000 | p =0.162 | 1.00 ± 0.000 | | 1.00 ± 0.000 | - | p =0.911 | p =0.185 |
| Write a sentence | 0.91 ± 0.283 | 0.81 ± 0.393 | **p =0.020*** | 0.80 ± 0.408 | 0.68 ± 0.478 | p =0.345 | 0.92 ± 0.277 | 0.95 ± 0.223 | p =0.559 | 1.00 ± 0.000 | | 1.00 ± 0.000 | - | p =0.030 | p =0.390 |
| Memory | 2.49 ± 0.761 | 2.59 ± 0.660 | p =0.380 | 2.36 ± 0.780 | 2.53 ± 0.612 | p =0.530 | 2.20 ± 0.928 | 2.26 ± 0.818 | p =0.924 | 2.00 ± 0.816 | | 2.67 ± 0.516 | p =0.157 | p =0.372 | p =0.780 |
| Visual construction | 0.89 ± 0.314 | 0.89 ± 0.310 | p =0.912 | 0.89 ± 0.321 | 0.89 ± 0.315 | p =0.923 | 0.80 ± 0.401 | 0.87 ± 0.339 | p =0.376 | 0.75 ± 0.500 | | 1.00 ± 0.000 | p =0.221 | p =0.718 | p =0.431 |
| Comprehension | 2.72 ± 0.730 | 2.56 ± 0.879 | p =0.111 | 2.91 ± 0.291 | 2.89 ± 0.315 | p =0.860 | 2.61 ± 0.842 | 2.64 ± 0.778 | p =0.965 | 1.50 ± 1.732 | | 2.50 ± 0.837 | p =0.335 | p =0.102 | p =0.118 |
| MoCA | 25.10 ± 3.865 | 24.54 ± 3.835 | p =0.224 | 26.27 ± 3.06 | 25.05 ± 3.223 | p =0.173 | 23.20 ± 4.304 | 24.05 ± 3.363 | p =0.450 | 23.00 ± 4.082 | | 24.83 ± 3.061 | p =0.438 | p =0.144 | p =0.170 |
| Orientation | 5.71 ± 0.731 | 5.82 ± 0.467 | p =0.434 | 5.95 ± 0.211 | 5.95 ± 0.229 | p =0.903 | 5.75 ± 0.767 | 5.90 ± 0.384 | p =0.290 | 5.50 ± 0.577 | | 5.83 ± 0.408 | p =0.285 | p =0.167 | p =0.874 |
| Attention | 5.65 ± 0.707 | 5.55 ± 0.764 | p =0.288 | 5.77 ± 0.476 | 5.68 ± 0.478 | p =0.378 | 5.43 ± 0.694 | 5.56 ± 0.754 | p =0.166 | 5.75 ± 0.500 | | 5.33 ± 0.816 | p =0.393 | p =0.262 | p =0.270 |
| Memory | 2.53 ± 1.745 | 2.26 ± 1.767 | p =0.251 | 2.75 ± 1.767 | 2.16 ± 1.834 | p =0.243 | 2.26 ± 1.769 | 2.15 ± 1.679 | p =0.770 | 2.00 ± 2.309 | | 2.83 ± 1.722 | p =0.392 | p =0.155 | p =0.789 |
| Language | 2.43 ± 0.765 | 2.22 ± 0.792 | **p =0.022*** | 2.43 ± 0.759 | 2.37 ± 0.684 | p =0.586 | 1.95 ± 0.884 | 2.33 ± 0.737 | p =0.029 | 1.25 ± 0.957 | | 2.50 ± 0.837 | p =0.060 | **p =0.022*** | **p <0.001*** |
| Naming | 2.65 ± 0.664 | 2.61 ± 0.725 | p =0.874 | 2.86 ± 0.347 | 2.63 ± 0.597 | p =0.086 | 2.36 ± 0.913 | 2.54 ± 0.72 | p =0.398 | 3.00 ± 0.000 | | 2.67 ± 0.816 | p =0.529 | p =0.831 | p =0.246 |
| Visual construction | 4.35 ± 0.952 | 4.18 ± 0.941 | p =0.079 | 4.57 ± 0.661 | 4.21 ± 0.918 | p =0.099 | 3.80 ± 1.352 | 3.90 ± 0.968 | p =0.764 | 4.00 ± 0.816 | | 4.50 ± 0.837 | p =0.298 | p =0.121 | p =0.320 |
| Abstract concepts | 1.27 ± 0.801 | 1.31 ± 0.802 | p =0.663 | 1.43 ± 0.661 | 1.37 ± 0.684 | p =0.720 | 0.93 ± 0.834 | 0.82 ± 0.79 | p =0.513 | 0.75 ± 0.500 | | 0.83 ± 0.753 | p =0.903 | p =0.620 | p =0.924 |
| ** p<0.025* | | | | | | | | | | |  | | | | |

| **Supplementary Table 4.** Cognition functions of participants in HBV, HCV, and HBV and HCV coinfection groups stratified by rs10417924 genotypes. | | | | | | | | | | | | | | | |
| --- | --- | --- | --- | --- | --- | --- | --- | --- | --- | --- | --- | --- | --- | --- | --- |
| Study Group | Normal Control (n = 258) | | | HBV (n = 63 ) | | | HCV (n = 100 ) | | | HBV + HCV coinfection (n = 10 ) | | | | Moderation analysis | |
| Genotype | TT/TC  (n = 64) | CC  (n = 194) | Statistic *p-value* | TT/TC  (n = 11) | CC  (n = 52) | Statistic  p-value | TT/TC  (n = 25) | CC  (n = 75) | Statistic  p-value | TT/TC  (n = 4) | | CC  (n = 6) | Statistic  p-value | SNP  effect | Moderation  effect |
| Age (years) | 65.75 ± 6.749 | 64.90 ± 7.132 | p = 0.401 | 59.82 ± 6.113 | 60.29 ± 6.298 | p =0.822 | 64.00 ± 8.431 | 62.52 ± 7.698 | p =0.379 | 52.5 ± 4.359 | | 66.5 ± 7.287 | p =0.005 |  |  |
| M/F | 14 / 50 | 55 / 139 | p = 0.310 | 7 / 4 | 37 / 15 | p =0.721 | 11 / 14 | 23 / 52 | p =0.234 | 1 / 3 | | 5 / 1 | p =0.191 |  |  |
| Education (years) | 12.02 ± 3.982 | 12.39 ± 3.986 | p = 0.519 | 13.64 ± 4.007 | 13.03 ± 3.185 | p =0.586 | 11.64 ± 3.377 | 10.31 ± 3.624 | p =0.061 | 13.75 ± 8.261 | | 12.67 ± 3.724 | p =0.782 |  |  |
| MMSE | 27.64 ± 1.863 | 26.94 ± 2.953 | p =0.143 | 28.45 ± 1.368 | 28.00 ± 1.442 | p =0.351 | 27.24 ± 2.107 | 26.99 ± 2.883 | p =0.897 | 26.25 ± 4.992 | | 26.17 ± 1.329 | p =0.976 | **p =0.014*** | p =0.351 |
| Orientation to time | 4.83 ± 0.606 | 4.58 ± 0.786 | **p =0.002*** | 4.82 ± 0.405 | 4.92 ± 0.334 | p =0.182 | 4.56 ± 0.651 | 4.64 ± 0.710 | p =0.400 | 4.75 ± 0.500 | | 4.50 ± 0.548 | p =0.453 | **p =0.009*** | p =0.124 |
| Orientation to place | 4.11 ± 0.715 | 4.12 ± 0.871 | p =0.599 | 4.82 ± 0.405 | 4.79 ± 0.412 | p =0.826 | 4.52 ± 0.586 | 4.60 ± 0.697 | p =0.331 | 4.25 ± 0.957 | | 4.83 ± 0.408 | p =0.236 | p =0.990 | p =0.578 |
| Registration | 3.00 ± 0.000 | 2.97 ± 0.189 | p =0.248 | 3.00 ± 0.000 | 3.00 ± 0.000 | - | 2.96 ± 0.200 | 2.96 ± 0.197 | p =1.000 | 3.00 ± 0.000 | | 3.00 ± 0.000 | - | p =0.201 | p =0.765 |
| Serial 7's | 4.70 ± 0.525 | 4.46 ± 0.900 | p =0.194 | 4.64 ± 0.505 | 4.42 ± 0.750 | p =0.497 | 4.48 ± 0.823 | 4.31 ± 1.039 | p =0.491 | 3.75 ± 1.893 | | 3.67 ± 1.033 | p =0.930 | p =0.032 | p =0.868 |
| Naming | 2.00 ± 0.000 | 2.00 ± 0.000 | - | 2.00 ± 0.000 | 2.00 ± 0.000 | - | 2.00 ± 0.000 | 2.00 ± 0.000 | - | 2.00 ± 0.000 | | 2.00 ± 0.000 | - | - | - |
| Repetition | 0.94 ± 0.244 | 0.94 ± 0.232 | p =0.864 | 1.00 ± 0.000 | 0.96 ± 0.194 | p =0.512 | 0.92 ± 0.277 | 0.99 ± 0.115 | p =0.092 | 0.75 ± 0.500 | | 1.00 ± 0.000 | p =0.221 | p =0.957 | p =0.087 |
| Read and obey command | 0.94 ± 0.244 | 0.96 ± 0.199 | p =0.485 | 0.91 ± 0.302 | 1.00 ± 0.000 | p =0.030 | 1.00 ± 0.000 | 0.96 ± 0.197 | p =0.312 | 1.00 ± 0.000 | | 1.00 ± 0.000 | - | p =0.500 | p =0.193 |
| Write a sentence | 0.89 ± 0.315 | 0.88 ± 0.330 | p =0.760 | 0.82 ± 0.405 | 0.75 ± 0.437 | p =0.632 | 0.96 ± 0.200 | 0.92 ± 0.273 | p =0.499 | 1.00 ± 0.000 | | 1.00 ± 0.000 | - | p =0.626 | p =0.996 |
| Memory | 2.53 ± 0.689 | 2.52 ± 0.743 | p =0.889 | 2.64 ± 0.674 | 2.37 ± 0.742 | p =0.213 | 2.12 ± 0.971 | 2.25 ± 0.856 | p =0.601 | 2.50 ± 0.577 | | 2.33 ± 0.816 | p =0.814 | p =0.764 | p =0.567 |
| Visual construction | 0.91 ± 0.294 | 0.89 ± 0.318 | p =0.662 | 0.82 ± 0.405 | 0.90 ± 0.298 | p =0.415 | 0.92 ± 0.277 | 0.80 ± 0.403 | p =0.169 | 0.75 ± 0.500 | | 1.00 ± 0.000 | p =0.221 | p =0.618 | p =0.349 |
| Comprehension | 2.80 ± 0.540 | 2.62 ± 0.844 | p =0.247 | 3.00 ± 0.000 | 2.88 ± 0.323 | p =0.240 | 2.80 ± 0.645 | 2.56 ± 0.858 | p =0.135 | 2.50 ± 1.000 | | 1.83 ± 1.472 | p =0.399 | p =0.057 | p =0.939 |
| MoCA | 24.67 ± 3.797 | 24.99 ± 3.873 | p =0.441 | 26.73 ± 2.901 | 25.73 ± 3.182 | p =0.312 | 24.48 ± 2.786 | 23.21 ± 4.259 | p =0.358 | 25.25 ± 3.862 | | 23.33 ± 3.204 | p =0.416 | p =0.716 | p =0.613 |
| Orientation | 5.86 ± 0.467 | 5.71 ± 0.706 | p =0.090 | 6.00 ± 0.000 | 5.94 ± 0.235 | p =0.418 | 5.84 ± 0.473 | 5.80 ± 0.697 | p =0.994 | 6.00 ± 0.000 | | 5.50 ± 0.548 | p =0.109 | p =0.069 | p =0.751 |
| Attention | 5.66 ± 0.718 | 5.60 ± 0.729 | p =0.447 | 5.64 ± 0.674 | 5.77 ± 0.425 | p =0.659 | 5.48 ± 0.586 | 5.48 ± 0.760 | p =0.629 | 5.25 ± 0.957 | | 5.67 ± 0.516 | p =0.464 | p =0.459 | p =0.259 |
| Memory | 2.22 ± 1.804 | 2.51 ± 1.731 | p =0.302 | 3.55 ± 1.036 | 2.37 ± 1.858 | p =0.061 | 2.40 ± 1.633 | 2.16 ± 1.763 | p =0.601 | 4.00 ± 0.816 | | 1.50 ± 1.761 | **p =0.018*** | p =0.277 | p =0.073 |
| Language | 2.25 ± 0.797 | 2.40 ± 0.771 | p =0.127 | 2.82 ± 0.405 | 2.33 ± 0.760 | p =0.036 | 2.12 ± 0.881 | 2.09 ± 0.841 | p =0.846 | 1.75 ± 0.957 | | 2.17 ± 1.169 | p =0.572 | p =0.195 | p =0.059 |
| Naming | 2.64 ± 0.698 | 2.63 ± 0.680 | p =0.868 | 2.64 ± 0.674 | 2.83 ± 0.382 | p =0.381 | 2.76 ± 0.436 | 2.32 ± 0.918 | p =0.043 | 2.50 ± 1.000 | | 3.00 ± 0.000 | p =0.221 | p =0.621 | p =0.071 |
| Visual construction | 4.31 ± 0.924 | 4.28 ± 0.959 | p =0.857 | 4.27 ± 1.009 | 4.50 ± 0.700 | p =0.583 | 4.20 ± 0.645 | 3.72 ± 1.331 | p =0.253 | 4.25 ± 0.957 | | 4.33 ± 0.816 | p =0.908 | p =0.511 | p =0.153 |
| Abstract concepts | 1.19 ± 0.833 | 1.31 ± 0.788 | p =0.306 | 1.36 ± 0.809 | 1.42 ± 0.637 | p =0.952 | 1.00 ± 0.816 | 0.85 ± 0.817 | p =0.428 | 1.00 ± 0.816 | | 0.67 ± 0.516 | p =0.464 | p =0.346 | p =0.860 |
| ** p<0.025* | | | | | | | | | | |  | | | | |
